# Pro-EGCG suppresses endometriosis progression via regulating monocytic myeloid-derived suppressor cells

**DOI:** 10.1186/s13020-026-01451-8

**Published:** 2026-07-01

**Authors:** Qianhan Xu, Lu Chen, Jun Chen, Haoyue Hu, Zhihua Luo, Zhentao Gong, Yee Lee Ng, Jinchuan Liu, Sze Wan Hung, Yi Song, Nga Ping Ip, See Yung Chau, Yonggang Duan, Chi Chiu Wang, Ting Guo, Kam Tong Leung, Jingying Zhou, Pui Wah Chung, Tao Zhang

**Affiliations:** 1 Assisted Reproductive Technology Unit, Department of Obstetrics and Gynaecology, Faculty of Medicine, The Chinese University of Hong Kong, Hong Kong, China; 2 Department of Obstetrics and Gynecology, Nanfang Hospital, Southern Medical University, Guangzhou, Guangdong China; 3 Reproductive Medicine Center, The Eighth Affiliated Hospital, Southern Medical University (The First People’s Hospital of Shunde), Foshan, Guangdong China; 4 Department of Gynaecology, Shenzhen Maternity and Child Healthcare Hospital, Southern Medical University, Shenzhen, Guangdong China; 5 Shenzhen Key Laboratory of Fertility Regulation, Center of Assisted Reproduction and Embryology, The University of Hong Kong - Shenzhen Hospital, Shenzhen, Guangdong China; 6 School of Biomedical Sciences, The Chinese University of Hong Kong, Hong Kong, China; 7 Reproduction and Development Laboratory, Li Ka Shing Institute of Health Sciences, The Chinese University of Hong Kong, Hong Kong, China; 8 Chinese University of Hong Kong - Sichuan University Joint Laboratory in Reproductive Medicine, The Chinese University of Hong Kong, Hong Kong, China; 9 State Key Laboratory of Reproductive Medicine and Offspring Health, Center for Reproductive Medicine, Institute of Women, Children and Reproductive Health, Shandong University, Shandong, China; 10 Hospital for Reproductive Medicine Affiliated to Shandong University, Shandong, China; 11 Department of Pediatrics, Prince of Wales Hospital, The Chinese University of Hong Kong, Hong Kong, China

**Keywords:** Pro-EGCG, Myeloid-derived suppressor cells, Endometriosis, Monocytic MDSCs, Immunosuppression, Fibrosis

## Abstract

**Background:**

Pro-EGCG has shown therapeutic promise for endometriosis, yet its immunoregulatory mechanisms remain unclear. Myeloid-derived suppressor cells (MDSCs) are key drivers of disease progression. This study investigates whether Pro-EGCG alleviates endometriosis by modulating MDSC-mediated immune and stromal dysregulation.

**Methods:**

The therapeutic effects of Pro-EGCG were evaluated in an experimental endometriosis mouse model by assessing lesion burden, histology, and MDSC dynamics. The functional necessity of monocytic MDSCs (M-MDSCs) was validated via adoptive transfer. Clinical relevance was assessed in peripheral blood and lesions from women with or without endometriosis via flow cytometry and multiplex immunofluorescence staining. Furthermore, the direct effects of Pro-EGCG on human PBMC-derived M-MDSCs were examined *in vitro*, including their immunosuppressive function and their ability to promote fibrosis in a co-culture system with human endometriotic stromal cells.

**Results:**

In the mouse model, Pro-EGCG treatment significantly reduced lesion weight, volume, and fibrosis, accompanied by consistent M-MDSC reduction systemically and locally. Lesion-infiltrating M-MDSCs, rather than polymorphonuclear MDSCs (PMN-MDSCs), positively correlated with disease severity. Adoptive transfer of M-MDSCs reversed Pro-EGCG’s therapeutic effects. In clinical samples, data confirmed a significant expansion of M-MDSCs in patients with endometriosis compared to controls. *In vitro*, Pro-EGCG compromised human M-MDSC survival and impaired their suppressive capacity by inhibiting ROS, Arg-1, and NO production. Furthermore, M-MDSC-induced stromal cell proliferation and fibrotic gene expression were abolished by Pro-EGCG preconditioning.

**Conclusion:**

Pro-EGCG hinders endometriosis progression by inhibiting M-MDSC accumulation, immunosuppressive functions, and pro-endometriotic activities. These findings position Pro-EGCG as a potential immunotherapy for endometriosis and other M-MDSC-driven inflammatory disorders.

**Supplementary Information:**

The online version contains supplementary material available at 10.1186/s13020-026-01451-8.

## Introduction

Endometriosis is an estrogen-dependent inflammatory disorder characterized by the presence of endometrial-like tissue outside the uterine cavity [1]. It affects approximately 190 million women worldwide and is a major cause of dysmenorrhea, chronic pelvic pain, and infertility [2]. Current treatment options remain limited and noncurative, which severely compromises patients’ quality of life and is frequently associated with psychological stress, depression, and family instability [3]. Long-term disease management also places a substantial burden on healthcare systems and leads to considerable financial costs for affected women and society [2]. These challenges highlight the need for novel, effective, and precise therapeutic strategies that target the underlying pathogenesis of endometriosis.

In recent decades, efforts to move beyond traditional hormonal suppression have prompted the exploration of alternative therapeutic approaches, including the use of natural products [4, 5]. We previously reported, for the first time, the therapeutic effects of a prodrug of epigallocatechin-3-gallate (Pro-EGCG) in endometriosis [6, 7]. Pro-EGCG is a synthetic derivative of EGCG, a polyphenol found in green tea, and exhibits superior stability and bioavailability compared with the parent compound [7, 8]. Our earlier studies demonstrated that Pro-EGCG significantly suppresses the development of endometriotic lesions by reducing endometriotic cell proliferation and angiogenesis, and that it outperforms both EGCG and vehicle controls [6, 7]. However, the effect of Pro-EGCG on the immunosuppressive microenvironment that supports endometriosis development remains entirely unclear.

Immunosuppression is a characteristic feature of endometriosis, and accumulating evidence underscores the central role of immune dysregulation in its pathophysiological progression [9]. Impaired cytotoxicity of natural killer (NK) cells, dysfunctional macrophages, and imbalanced T-cell subsets within the peritoneal fluid (PF) collectively compromise immune surveillance [10]. This immune dysfunction allows for the persistence of ectopic endometrial cells, fosters angiogenesis and promotes fibrosis [10]. Although the precise mechanisms underlying these alterations are not fully elucidated, our previous work suggests that myeloid-derived suppressor cells (MDSCs), which accumulate dramatically at the onset of endometriosis, are key contributors to local immunosuppression and neovascularization within the endometriotic lesions [11].

MDSCs are a heterogeneous population of immature myeloid cells with potent immunosuppressive activity, classically expanding in cancer and chronic inflammatory conditions [12]. They are conventionally divided into two main subsets: polymorphonuclear MDSCs (PMN-MDSCs), which are phenotypically and functionally similar to neutrophils, and monocytic MDSCs (M-MDSCs), which share features with monocytes [12]. Although both subsets exert immunosuppressive functions, M-MDSCs are increasingly recognized in inflammatory diseases as a particularly potent subset. They suppress T-cell responses and are associated with upregulation of arginase-1 (Arg-1), nitric oxide (NO), reactive oxygen species (ROS), and immune checkpoint molecules such as programmed death-ligand 1 (PD-L1) [13, 14]. Evidence further indicates that M-MDSCs possess a broader repertoire of regulatory functions and greater phenotypic plasticity than PMN-MDSCs [15, 16]. Beyond immunosuppression, findings from cancer research demonstrate that MDSCs also contribute to disease progression through non-immune mechanisms, including directly promoting tumor cell proliferation, migration, and invasion, as well as facilitating fibrosis and tissue remodeling by modulating stromal cell activity within the pathological microenvironment [17, 18]. Together, these multifaceted functions highlight the capacity of MDSCs to influence diverse cellular populations within the pathological microenvironment of diseased tissues and underscore the increasing attention toward M-MDSCs in studies of immune dysregulation across chronic inflammatory disorders.

In the present study, we therefore aimed to investigate the effect of Pro-EGCG on MDSCs in endometriosis. Using a combination of *in vivo *therapeutic experiments, clinical sample analyses, and *in vitro* models, we demonstrate that Pro-EGCG effectively attenuates the accumulation and immunosuppressive activity of M-MDSCs. This action restores immune surveillance and counteracts the pro-endometriotic effects driven by M-MDSCs.

## Materials and methods

### Murine endometriosis model and Pro-EGCG treatment

All animal experiments were approved by the Institutional Animal Care and Use Committee (IACUC, protocol no. APS-250221-028-01) and were conducted in accordance with national guidelines for the care and use of laboratory animals. A syngeneic endometriosis model was established in female C57BL/6 mice (6–8 weeks old) using a previously described surgical procedure [19, 20]. To synchronize estrous cycles, a single intramuscular injection of estradiol was administered three days prior to surgery. Briefly, the left uterine horn was excised under sterile conditions, cut into approximately 2 mm fragments, and 4 fragments were sutured onto the peritoneal wall close to blood vessels in the same mouse to generate endometrial implants.

Four weeks after induction, mice were randomly assigned to two groups: a vehicle group (n = 8) and a Pro-EGCG group (n = 8). The treatment group received Pro-EGCG at 50 mg/kg/day by oral gavage for four consecutive weeks, whereas control mice received an equal volume of vehicle (olive oil). The Pro-EGCG dose of 50 mg/kg was selected based on its pharmacokinetic profile [21] and prior efficacy in an endometriosis model [22]. At the end of the 4-week treatment period, endometriotic lesions were carefully excised. Lesion volume was calculated using the formula: volume = (length × width × height × π/6). Lesion weight was recorded after blotting away excess fluid. At the experimental endpoint, endometriotic lesions, bone marrow (BM), peripheral blood (PB), and PF were collected for histological and flow cytometric analyses.

### Adoptive transfer experiment

For adoptive transfer of MDSCs to Pro-EGCG-treated mice with endometriosis, BM was collected from endometriosis-bearing donor C57BL/6 mice at 4 weeks post-induction. Single-cell suspensions were prepared, erythrocytes were lysed, and nucleated cells were stained with antibodies against CD11b, Ly6G, and Ly6C. M-MDSCs (CD11b^+^Ly6G^−^Ly6C^+^) were sorted at > 90% purity using a BD FACSAria cell sorter and resuspended in RPMI-1640 medium supplemented with 10% fetal bovine serum (FBS) at a concentration of 2 × 10^6^ cells in 100 µL [23, 24].

Recipient mice with established endometriosis (4 weeks post-induction) were randomly assigned to three groups (n = 10 per group): (1) Vehicle, (2) Pro-EGCG, and (3) Pro-EGCG + M-MDSCs. Starting on day 28 post-induction, mice in groups 2 and 3 received daily oral gavage of Pro-EGCG (50 mg/kg body weight in 100µL olive oil), while mice in group 1 received vehicle alone (100µL olive oil) daily. Beginning on the same day (day 28), mice in group 3 additionally received weekly retro-orbital injections of sorted M-MDSCs under isoflurane anesthesia for four consecutive weeks (on days 28, 35, 42, 49) [23, 24]. All treatments were continued until the experimental endpoint. Lesion burden and MDSC populations in BM, PB, PF, and lesions were analyzed at the endpoint.

### Human samples and ethical approval

Peripheral blood samples were obtained from women with laparoscopically confirmed endometriosis (n = 20) and from control women without endometriosis (n = 30) at the Department of Obstetrics and Gynaecology, Prince of Wales Hospital, The Chinese University of Hong Kong. Control women underwent laparoscopy for benign gynaecological indications unrelated to endometriosis, including unexplained infertility (n = 13), tubal factor disease (n = 11), non-endometriotic ovarian cyst (n = 4), and polycystic ovary syndrome (n = 2). Absence of endometriosis was confirmed by direct laparoscopic visualization in all control participants. None of the participants had received hormonal treatment in the 3 months preceding sample collection. Written informed consent was obtained from all participants. This study was approved by the Joint Chinese University of Hong Kong-New Territories East Cluster Clinical Research Ethics Committee (CREC2022.163).

### Identification and characterization of MDSCs in mouse and human samples by flow cytometry

Single-cell suspensions were prepared from mouse BM, PB, and PF to analyze mouse MDSCs. All murine samples were processed fresh within 2 h of collection and were not cryopreserved prior to staining. For murine peripheral blood, samples were processed by direct red blood cell lysis (RBC) of whole blood without density gradient centrifugation, to preserve both PMN-MDSC and M-MDSC populations. After RBC lysis and Fc receptor blocking, cells were stained with fluorochrome-conjugated antibodies against 7-AAD, CD45, CD11b, Ly6G, and Ly6C. Samples were acquired on a BD LSRFortessa cell analyzer, and data were analyzed using FlowJo software (Version 10.8.1). The gating strategy involved sequential identification of single, live cells, followed by selection of the CD11b⁺ myeloid cell population. Within this gate, PMN-MDSCs and M-MDSCs were defined as CD11b⁺Ly6G⁺Ly6C⁻ and CD11b⁺Ly6G⁻Ly6C⁺ cells, respectively (Additional file 1: Fig. S1) [12].

Peripheral blood mononuclear cells (PBMCs) were isolated from human blood samples by Ficoll-Paque PLUS (GE Healthcare) density gradient centrifugation for the analysis of human MDSCs. All human samples were processed fresh within 2 h of collection and were not cryopreserved prior to staining. After Fc receptor blocking, cells were stained with fluorochrome-conjugated monoclonal antibodies against CD45, HLA-DR, CD33, CD11b, CD14, CD15, and PD-L1, with 7-AAD used to exclude dead cells. Flow cytometry acquisition and analysis were performed as described above. Total MDSCs were identified as CD45^+^HLA-DR^−^CD11b^+^CD33^+^ cells. PMN-MDSCs and M-MDSCs were subsequently defined as CD15^+^CD14^−^ and CD14^+^CD15^−^ subsets, respectively, within the total MDSC gate [12, 13]. Frequencies were expressed as a percentage of live CD45^+^ leukocytes, and PD-L1 positivity was evaluated within each subset. Detailed information on all fluorescence-conjugated antibodies used is provided in Additional file 1: Table S1.

### Histological analysis of endometriotic lesions in mice

Endometriotic lesions were fixed in 10% neutral-buffered formalin and embedded in paraffin. Sections were stained with hematoxylin and eosin (H&E) for general histopathological evaluation. Ki-67 immunohistochemistry was performed to assess cell proliferation. Apoptosis was examined using a TUNEL assay, and the apoptotic index was calculated as the percentage of TUNEL⁺ cells among total nucleated cells within stromal regions. Masson's trichrome staining was used to assess collagen deposition and fibrosis, and the fibrotic area (blue staining) was quantified as a percentage of the total lesion area using image analysis software. Immunohistochemical staining for α-smooth muscle actin (α-SMA) was performed to evaluate stromal fibrotic activation.

### Multiplex immunofluorescence staining of MDSCs in endometriotic lesions

Multiplex immunofluorescence staining was performed on paraffin-embedded mouse and human endometriotic lesion sections using the Opal multiplex immunofluorescence kit according to the manufacturer’s protocol. Primary antibodies were applied sequentially with tyramide signal amplification. For mouse lesions, the following antibody-fluorophore combinations were used: CD11b (Opal 520), Ly6C (Opal 570), and Ly6G (Opal 620). For human lesions, a distinct panel was employed: CD11b (Opal 480), CD14 (Opal 520), and CD15 (Opal 570). Nuclei were counterstained with DAPI in all samples.

Multispectral images were acquired using the Mantra imaging system and analyzed with InForm image analysis software (Version 2.4.6). The software was used to define stromal region and segment individual cell nuclei (DAPI⁺). For mouse samples, PMN-MDSCs and M-MDSCs were defined as CD11b⁺Ly6G⁺Ly6C⁻ and CD11b⁺Ly6G⁻Ly6C⁺ cells, respectively. For human samples, PMN-MDSCs and M-MDSCs were identified as CD11b⁺CD14⁻CD15⁺ and CD11b⁺CD14⁺CD15⁻ cells, respectively. In both cases, the proportion of each MDSC subset was calculated as the cell number relative to the total number of stromal cells per high-power field. The mean proportion across five randomly selected fields was used as the value for each lesion in statistical analysis. Detailed information on all antibodies used for multiplex immunofluorescence staining is provided in Additional file 1: Table S1.

### *In vitro* differentiation and functional assays of human M-MDSCs

#### Differentiation of M-MDSCs from PBMCs

The *in vitro* induction of M-MDSCs from PBMCs was performed based on established protocols [25, 26]. In brief, PBMCs were cultured in RPMI-1640 medium supplemented with 10% FBS, 1% penicillin–streptomycin, and 2 mM l-glutamine. To induce M-MDSC differentiation, cells were stimulated with recombinant human GM-CSF (20 ng/mL, BioLegend) and IL-6 (20 ng/mL; Sigma-Aldrich) for 6 days. CD14^+^ M-MDSCs were then isolated by positive selection using magnetic microbeads (MojoSort™ human CD14 selection kit, BioLegend). Cell purity, confirmed by flow cytometry staining for CD33 and CD14, was consistently > 90% (Additional file 1: Fig. S2).

#### Pro-EGCG treatment and assessment of cell viability and apoptosis

Purified M-MDSCs were seeded in culture plates and treated with increasing concentrations of Pro-EGCG (0, 20, 40, and 60 μM) for 24 h. Cell viability was assessed using a Cell Counting Kit-8 (CCK-8, Abcam) according to the manufacturer's instructions. Apoptosis was determined by flow cytometry using Annexin V-FITC and 7-AAD staining.

#### Analysis of M-MDSC immunosuppressive activity

The immunosuppressive activity of M-MDSCs was evaluated by measuring the production or expression of key functional mediators. The expression of PD-L1 on M-MDSCs was analyzed by flow cytometry. Intracellular ROS levels were assessed by flow cytometry after staining with 2′,7′-dichlorofluorescin diacetate (DCF-DA; Thermo Scientific). NO levels in culture supernatants were quantified using a Griess reagent assay (Thermo Scientific). The enzymatic activity of Arg-1 in cell lysates was measured using a colorimetric assay kit (Sigma-Aldrich).

#### T-cell proliferation assay

A CFSE-based T-cell proliferation assay was used to evaluate M-MDSC immunosuppressive function [25]. Isolated human CD3⁺ T cells were labeled with CFSE. M-MDSCs were pretreated with Pro-EGCG (0–60 μM) for 24 h, washed thoroughly, and then co-cultured with CFSE-labeled T cells (1:1 ratio) in anti-CD3/CD28 pre-coated plates for 72 h. Control groups included unstimulated T cells (background proliferation), stimulated T cells alone (maximum proliferation), and stimulated T cells co-cultured with untreated M-MDSCs (baseline suppression). T-cell proliferation was analyzed by flow cytometry, and the suppression percentage was calculated as: Suppression (%) = [1 − (% proliferating T cells in co-culture/% proliferating T cells in stimulated control)] × 100.

### Co-culture of M-MDSCs with endometriotic stromal cells to assess pro-endometriosis capacity

To delineate the direct effects of M-MDSCs on the pathogenic behaviors of endometriotic stromal cells, the human endometriotic stromal cell line 12Z was co-cultured with untreated or Pro-EGCG-pretreated M-MDSCs (60 μM for 24 h). Proliferation and migration of 12Z cells were assessed by EdU incorporation and scratch wound healing assays, respectively. Cellular invasion was evaluated using a non-contact Transwell system (0.4 μm pore size; Corning), with 12Z cells seeded in Matrigel-coated upper chambers and M-MDSCs or Pro-EGCG-pretreated M-MDSCs in the lower chamber. After 24 h, invading cells were stained and counted.

### Quantitative real-time PCR (qRT-PCR)

After 24 h of co-culture, total RNA was extracted from 12Z cells using a commercial RNA extraction kit. cDNA was synthesized from 1 μg total RNA using the PrimeScript RT reagent kit. Quantitative real-time PCR (qRT-PCR) was subsequently performed on a QuantStudio 5 system using SYBR Green Master Mix. The reaction protocol consisted of an initial denaturation at 95 °C for 10 min, followed by 40 cycles of 95 °C for 15 s and 60 °C for 1 min.

The expression of fibrosis-associated marker genes (*TGF-β, ACTA2 (α-SMA), VIM, COL1A1, COL1A2*) was analyzed. All samples were run in triplicate, and mRNA levels were normalized to *ACTB (β-actin)* expression. Relative gene expression was calculated using the 2^–(ΔΔCT)^ method, with 12Z cells cultured alone as the calibrator. Primer sequences are provided in Additional file 1: Table S2.

### Statistical analysis

Data distribution was assessed for normality using the Shapiro–Wilk test. Data that passed the normality test are presented as mean ± standard deviation (SD) and were analyzed using parametric tests: comparisons between two groups were made with an unpaired two-tailed Student’s *t* test, and comparisons among multiple groups were made with one-way analysis of variance (ANOVA) followed by Tukey’s post-hoc test. For data that did not meet the assumption of normality, non-parametric tests (Mann–Whitney *U* test for two groups, Kruskal–Wallis test with Dunn’s post-hoc test for multiple groups) were employed, and data are presented accordingly. A *P* value < 0.05 was considered statistically significant. All analyses were conducted using GraphPad Prism (Version 10.2.3) and IBM SPSS (Version 26.0).

## Results

### Pro-EGCG treatment attenuates endometriotic lesion growth and histopathological severity

**Fig. 1:**
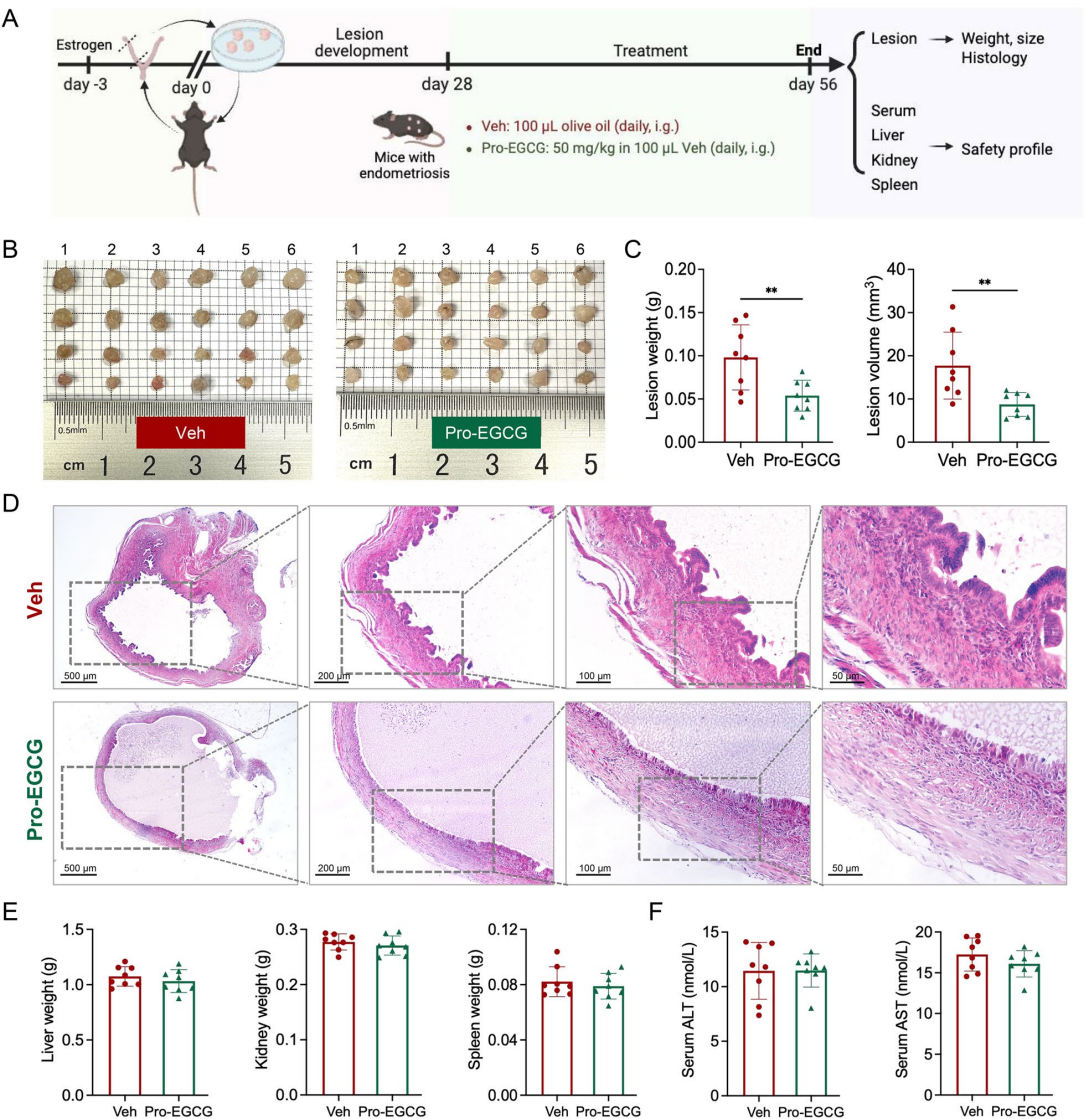
Pro-EGCG suppresses lesion progression and demonstrates biosafety in endometriosis mouse model. **A** Schematic diagram of the experimental timeline for surgically induced murine endometriosis and subsequent vehicle or Pro-EGCG treatment. **B** Representative gross morphology of endometriotic lesions harvested from vehicle- and Pro-EGCG-treated mice (6 mice per group shown; quantitative data are from all n = 8 animals per group). **C** Quantification of lesion weight and volume; data represent the mean value of 4 lesions per mouse (n = 8 mice per group). **D** Representative H&E staining of endometriotic lesions from vehicle- and Pro-EGCG-treated mice. Scale bars as indicated. **E** Organ weights (liver, kidney, and spleen) at the experimental endpoint. **F** Serum levels of liver enzymes (ALT and AST). Data are presented as mean ± SD. Statistical significance was determined by unpaired two-tailed Student’s *t* test. **P < 0.01; ***P < 0.001

The therapeutic efficacy of Pro-EGCG was first evaluated in a murine endometriosis model (Fig. 1A). Following 4 weeks of oral administration, Pro-EGCG treatment significantly reduced the endometriotic lesion burden, as demonstrated by substantial decreases in both lesion weight (*P* < 0.01) and volume (*P* < 0.001) (Fig. 1B, C). Histological evaluation via H&E staining corroborated these findings; while vehicle-treated lesions exhibited well-developed ectopic endometrial glands and thickened lesion walls, Pro-EGCG–treated mice displayed markedly reduced glandular structures, decreased endometrial stromal areas, and thinner lesion walls (Fig. 1D).

Importantly, Pro-EGCG treatment was well-tolerated with no overt systemic toxicity. Body weight, organ weights, and major organ histology remained comparable between groups (Fig. 1E, Additional file 1: Fig. S3), and serum levels of aspartate aminotransferase (AST) and alanine aminotransferase (ALT) showed no significant differences (Fig. 1F), confirming a favorable safety profile.

### Pro-EGCG treatment reduces the abundance and infiltration of M-MDSCs in endometriosis

**Fig. 2:**
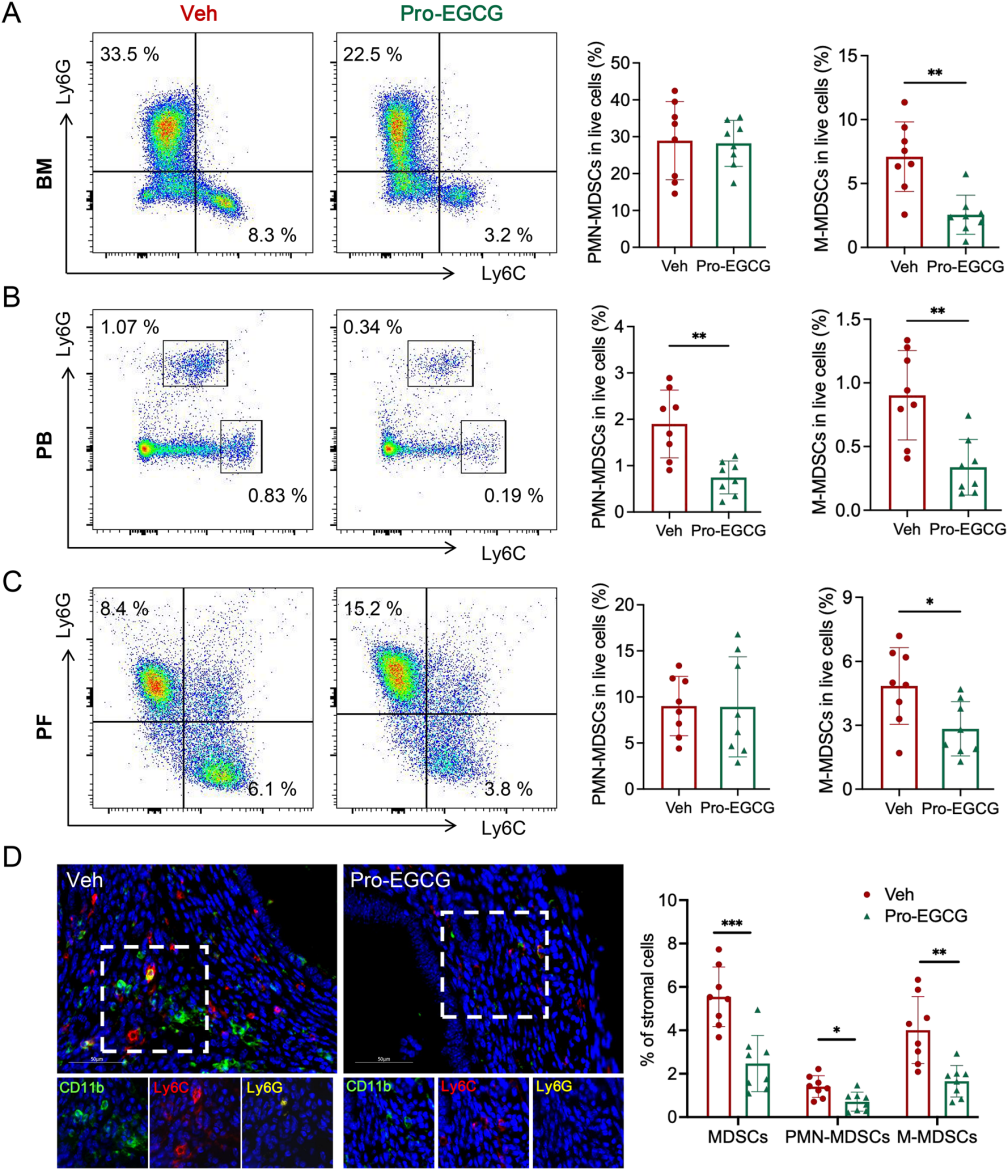
Pro-EGCG systemically and locally reduces M-MDSC accumulation across multiple compartments in endometriosis mouse model. **A**–**C** Representative flow cytometry plots used to identify PMN-MDSCs and M-MDSCs, bar graphs showing the frequencies of each subset among live cells in BM (**A**), PB (**B**), and PF (**C**) from vehicle- and Pro-EGCG-treated mice. **D** Representative multiplex immunofluorescence images and quantitative analysis of endometriotic lesions stained for CD11b (green), Ly6C (red), Ly6G (yellow), and DAPI (blue), showing infiltration of CD11b⁺ MDSCs, their subsets (Ly6G⁺ PMN-MDSCs, and Ly6C⁺ M-MDSCs). Data are presented as mean ± SD (n = 8 mice per group). Statistical significance was determined by unpaired two-tailed Student’s *t* test. *P < 0.05; **P < 0.01; ***P < 0.001; ns, not significant

To investigate whether Pro-EGCG alters the MDSC landscape, we examined their subset frequencies across multiple compartments. Pro-EGCG treatment significantly inhibited the M-MDSC subset systemically and locally. In the BM, the proportion of M-MDSCs significantly decreased following Pro-EGCG treatment (7.87% ± 2.08% vs. 5.52% ± 1.65%; *P* < 0.01; Fig. 2A). Similar reductions were observed in peripheral circulation (0.90% ± 0.35% vs. 0.34% ± 0.22%, *P* < 0.01; Fig. 2B) and within the peritoneal cavity (4.85% ± 1.80% vs. 2.84% ± 1.28%, *P* < 0.05; Fig. 2C). In contrast, PMN-MDSC reduction was only observed in the PB, but not in the BM or PF (Fig. 2A–C).

We further evaluated MDSC infiltration within endometriotic lesions using multiplex immunofluorescence. Consistent with systemic findings, the percentage of lesion-infiltrating M-MDSCs was significantly lower in the Pro-EGCG-treated group compared with vehicle group (4.01% ± 1.55% vs. 1.66% ± 0.73%; *P* < 0.01; Fig. 2D). A decrease in PMN-MDSC infiltration was also observed following Pro-EGCG treatment (1.41% ± 0.50% vs. 0.71% ± 0.44%; *P* < 0.05; Fig. 2D).

### Pathological severity of endometriotic lesions correlates with infiltrating M-MDSC abundance

**Fig. 3:**
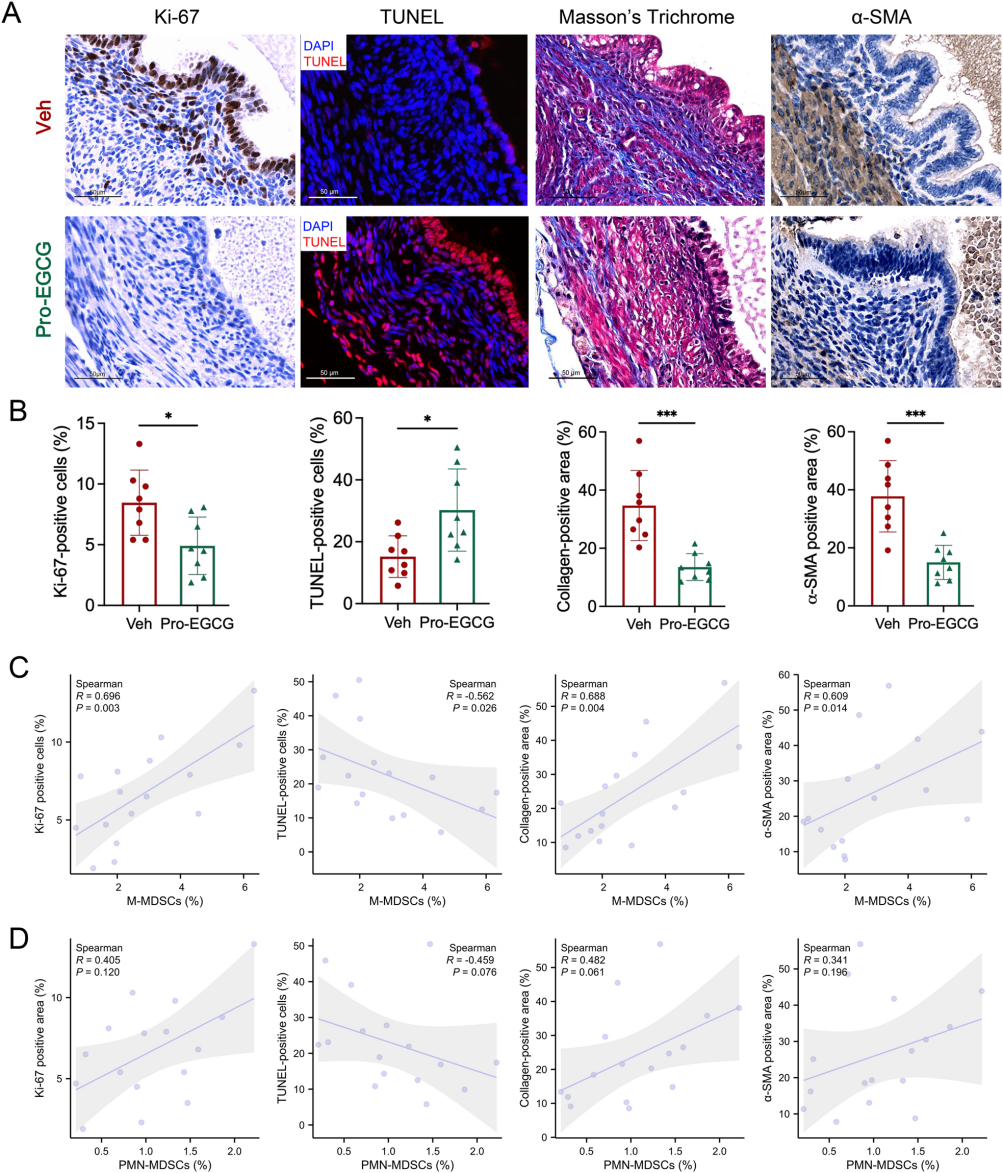
Pathological severity of endometriotic lesions correlates with infiltrating M-MDSC abundance. **A** Representative Masson’s trichrome, α-SMA, Ki-67, and TUNEL staining in endometriotic lesions from the indicated groups. **B** Quantification of Ki-67 positive cells, TUNEL-positive apoptotic cells, collagen-positive area, and α-SMA-positive area within endometriotic lesions. **C**, **D** Spearman correlation analyses between the frequencies of lesion-infiltrating M-MDSCs (**C**) or PMN-MDSCs (**D**) and histopathological parameters, including proliferation (Ki-67-positive cells), apoptosis (TUNEL-positive cells), as well as fibrosis (collagen-positive area and α-SMA-positive area) in individual mice. Spearman’s correlation coefficients (*R*) and *P* values are indicated. Data are presented as mean ± SD. Statistical significance was determined by unpaired two-tailed Student’s *t* test (**B**). *P < 0.05; **P < 0.01; ***P < 0.001

To explore the association between MDSC abundance and lesion pathogenicity, we evaluated key histopathological indices of disease severity. Pro-EGCG treatment significantly suppressed cellular proliferation within the lesions, indicated by a reduced Ki-67 index (8.46% ± 2.69% vs. 4.91% ± 2.36%; *P* < 0.05; Fig. 3A, B). Conversely, apoptotic activity was markedly enhanced, with TUNEL staining revealing an approximately twofold increase in apoptotic cells (15.18% ± 6.73% vs. 30.25% ± 13.28%; *P* < 0.05; Fig. 3A, B). Furthermore, Pro-EGCG treatment markedly attenuated fibrotic features, evidenced by a significant reduction in collagen-positive area via Masson’s trichrome staining (34.68% ± 12.09% vs. 13.50% ± 4.61%; *P* < 0.001) and a corresponding decrease in α-SMA expression (37.77% ± 12.28% vs. 15.01% ± 5.88%; *P* < 0.001; Fig. 3A, B).

Correlation analyses revealed that the frequency of lesion-infiltrating M-MDSCs positively correlated with the Ki-67 proliferation index (*R* = 0.696, *P* = 0.003), collagen-positive area (*R* = 0.688, *P* = 0.004), and α-SMA expression (*R* = 0.609, *P* = 0.014). In contrast, M-MDSC abundance was inversely correlated with the proportion of TUNEL-positive apoptotic cells (*R* = − 0.562, *P* = 0.026; Fig. 3C). Notably, no significant correlations were observed between lesion-infiltrating PMN-MDSCs and any of these histopathological indices (Fig. 3D). These findings suggest that M-MDSCs, rather than PMN-MDSCs, are more closely associated with proliferative and fibrotic progression in endometriotic lesions.

### Adoptive transfer of M-MDSCs abrogates the therapeutic benefits of Pro-EGCG

**Fig. 4:**
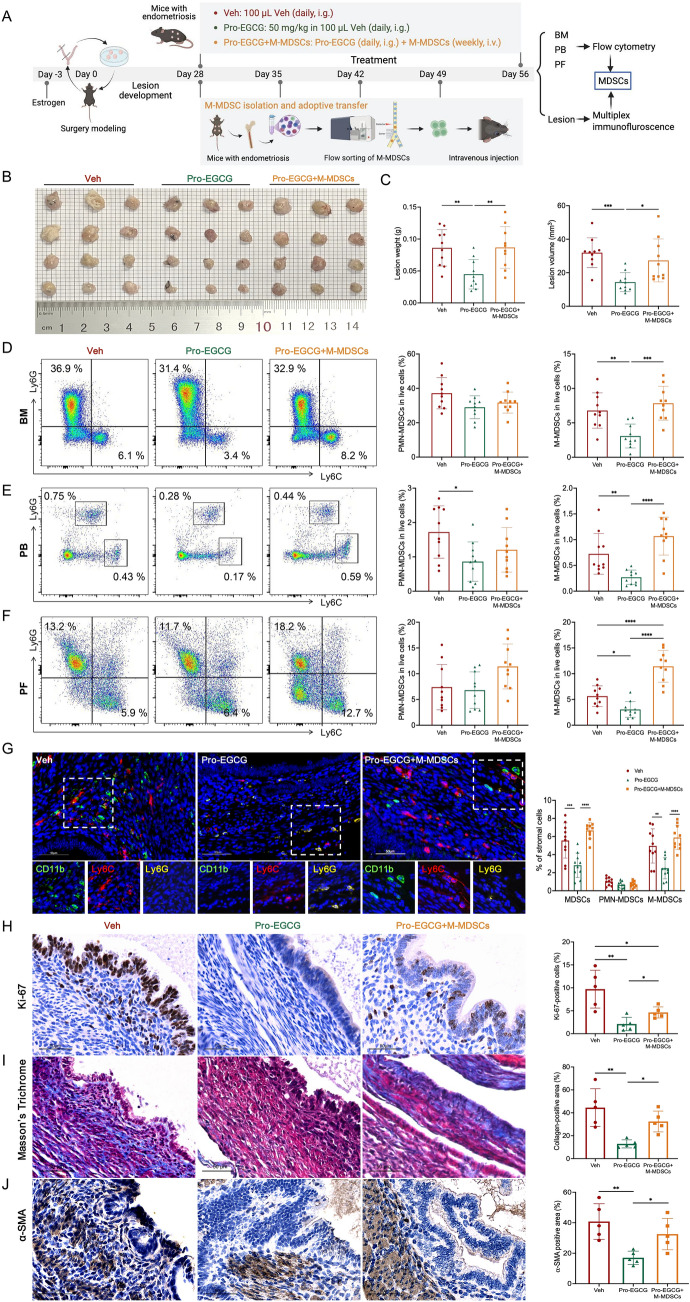
Adoptive transfer of M-MDSCs abrogates the therapeutic effects of Pro-EGCG against endometriosis. **A** Schematic diagram of the experimental design for M-MDSC adoptive transfer in Pro-EGCG-treated mice with endometriosis. **B** Representative gross morphology of endometriotic lesions from vehicle-treated, Pro-EGCG-treated, and Pro-EGCG plus M-MDSC adoptive transfer groups (6 mice per group shown; quantitative data are from all n = 10 animals per group). **C** Quantification of lesion weight and volume; data represent the mean value of 4 lesions per mouse (n = 10 mice per group). **D–F** Representative flow cytometry plots used to identify PMN-MDSCs and M-MDSCs, bar graphs showing the frequencies of each subset among live cells in BM (**D**), PB (**E**), and PF (**F**) from vehicle-treated, Pro-EGCG-treated, and Pro-EGCG-treated plus M-MDSC transfer groups (n = 10 mice per group). **G** Representative multiplex immunofluorescence images and quantitative analysis of endometriotic lesions stained for CD11b (green), Ly6C (red), Ly6G (yellow), and DAPI (blue), showing infiltration of total CD11b⁺ MDSCs, CD11b⁺ Ly6G⁺ PMN-MDSCs, and CD11b⁺ Ly6C⁺ M-MDSCs in the three groups (n = 10 mice per group). Scale bars as indicated. **H–J** Representative images and quantification of Ki-67 (**H**), collagen-positive area (**I**), and α-SMA-positive area (**J**) staining in endometriotic lesions. Data are presented as mean ± SD from 5 independent experiments. Statistical significance was determined by one-way ANOVA with Tukey’s post hoc test. *P < 0.05; **P < 0.01; ***P < 0.001; ****P < 0.0001

To investigate whether the reduction of M-MDSCs is essential for the therapeutic efficacy of Pro-EGCG, we performed restoration experiments by adoptively transferring M-MDSCs into Pro-EGCG-treated mice (Fig. 4A). M-MDSCs isolated from donor mice with endometriosis were confirmed for high purity (> 90%) and viability (Additional file 1: Fig. S4A). Successful systemic reconstitution was validated by the increase in circulating M-MDSC frequencies post-transfer (Additional file 1: Fig. S4B).

Phenotypic assessment revealed that restoration of the M-MDSC compartment markedly abolished the therapeutic benefits of Pro-EGCG. The reductions in lesion weight and volume achieved by Pro-EGCG were significantly reversed in Pro-EGCG-treated mice receiving M-MDSC transfer, returning to levels comparable to those observed in vehicle-treated controls (both *P* < 0.0001; Fig. 4B, C).

To determine whether this reversal of lesion burden was accompanied by restoration of the M-MDSC compartment, flow cytometric analyses were performed. In Pro-EGCG-treated mice receiving M-MDSC transfer, M-MDSC frequencies in the BM, PB, and PF were restored to levels comparable to those observed in vehicle-treated mice, and were significantly higher than those in Pro-EGCG-treated mice without transfer (Fig. 4D–F). Consistently, multiplex immunofluorescence analysis demonstrated that M-MDSC infiltration within endometriotic lesions was also restored following transfers (Fig. 4G).

Critically, the histopathological improvements mediated by Pro-EGCG, including reduced fibrosis as indicated by collagen deposition and α-SMA expression, as well as suppressed cellular proliferation, were effectively negated by M-MDSC reconstitution (Fig. 4H–J).

Together, these findings demonstrate that suppression of M-MDSCs is a prerequisite for the full therapeutic efficacy of Pro-EGCG in endometriosis.

### Pro-EGCG suppresses the survival and immunosuppressive properties of human M-MDSCs

**Fig. 5:**
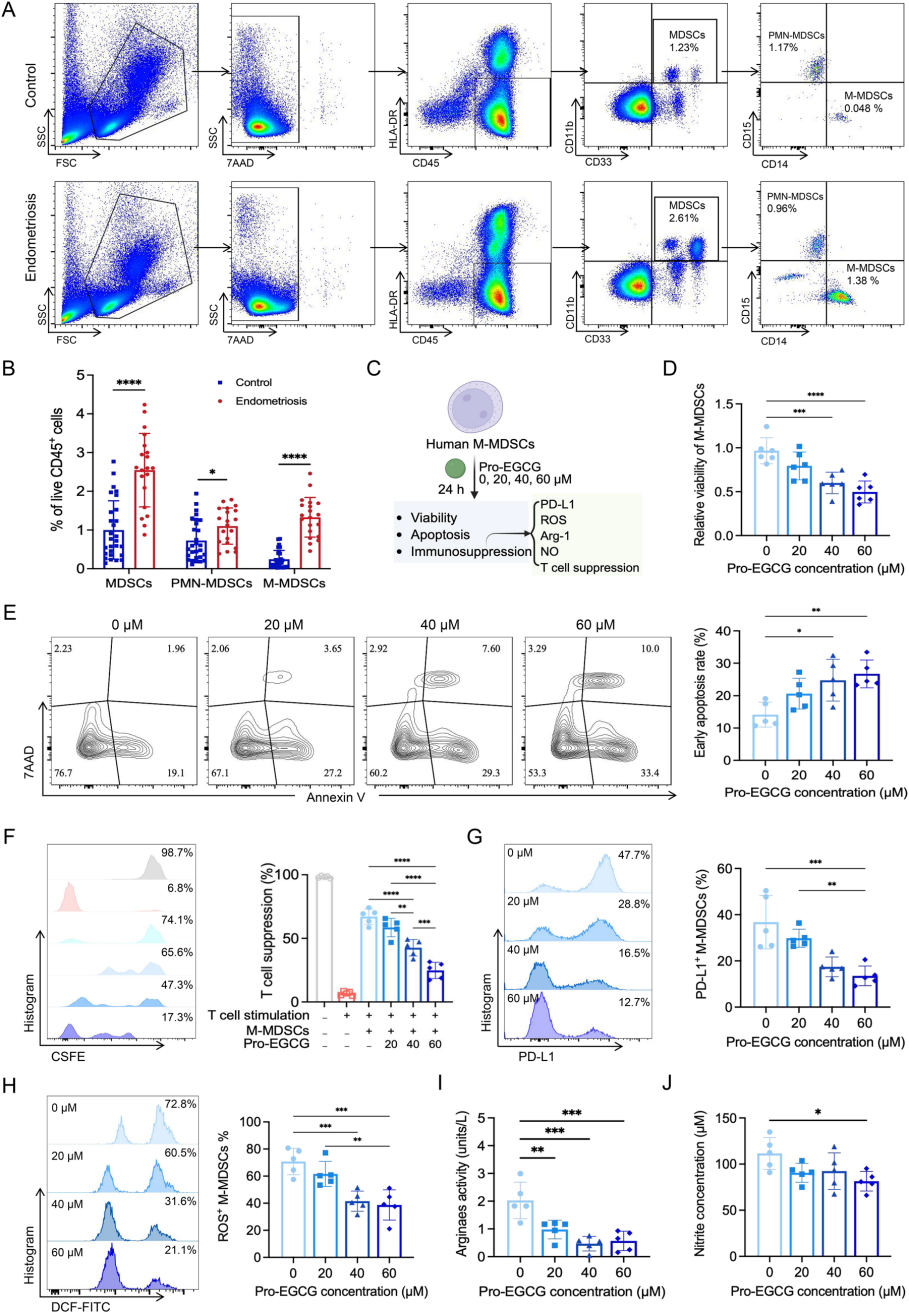
Pro-EGCG suppresses the immunosuppressive phenotypes and function of human M-MDSCs *in vitro*. **A** Representative flow cytometry plots of PBMCs from control individuals and endometriosis patients showing the gating strategy for total MDSCs (HLA-DR⁻CD33⁺CD11b⁺), PMN-MDSCs (HLA-DR⁻ CD33⁺ CD11b⁺ CD14⁻ CD15⁺), and M-MDSCs (HLA-DR⁻ CD33⁺ CD11b⁺ CD14⁺ CD15⁻). **B** Frequencies of total MDSCs, PMN-MDSCs, and M-MDSCs among live CD45^+^ peripheral blood cells from control individuals (n = 30) and endometriosis patients (n = 20). **C** Schematic diagram of the experimental design for assessing the direct effects of Pro-EGCG on human M-MDSCs* in vitro*. **D** Relative viability of M-MDSCs treated with Pro-EGCG, assessed by CCK-8 assay. **E** Representative Annexin V/7-AAD staining and quantification of apoptotic M-MDSCs following Pro-EGCG treatment. **F** Representative CFSE dilution profiles and quantification of T cell suppression in co-cultures with M-MDSCs with or without Pro-EGCG treatment. **G** Representative histograms and quantification of the frequency of PD-L1⁺ M-MDSCs after Pro-EGCG treatment. **H** Representative histograms and quantification of intracellular ROS levels (DCF-FITC) in M-MDSCs in response to Pro-EGCG. **I** Arg-1 activity in M-MDSCs following Pro-EGCG treatment. **J** NO production in M-MDSCs following Pro-EGCG treatment. Data are presented as mean ± SD from 5 to 6 independent experiments. Statistical significance was determined by unpaired two-tailed Student’s *t* test (**B**) and one-way ANOVA with Tukey’s post-hoc test (**D–J**). *P < 0.05; **P < 0.01; ***P < 0.001; ****P < 0.0001

To evaluate the clinical relevance of our findings, we first examined MDSC profiles in PB from women with endometriosis and non-endometriosis controls. Demographic characteristics were comparable between the two groups (Table 1). Flow cytometric analysis revealed a significant increase in the frequencies of total MDSCs in patients with endometriosis (2.61% ± 0.91% vs. 0.85% ± 0.52%; *P* < 0.0001; Fig. 5A, B), which was predominantly attributed to a marked expansion of the M-MDSC subset (1.90% ± 0.74% vs. 0.12% ± 0.13%; *P* < 0.0001; Fig. 5A, B).

**Table 1:** Clinical characteristics of endometriosis patients and control subjects

	Endometriosis	Control	*P* value
Age (years)	35.20 ± 6.22	35.12 ± 2.37	0.962
BMI (kg/m^2^)	21.78 ± 2.97	22.69 ± 3.04	0.399
Menstrual cycle length (days)	33.12 ± 5.62	29.93 ± 3.10	0.055
Menstrual duration (days)	6.00 ± 0.85	5.18 ± 1.50	0.063
Gravidity	0.60 ± 0.99	0.59 ± 0.87	0.972
Parity	0.47 ± 0.92	0.06 ± 0.24	0.114
Menstrual cycle phase at the time of sample collection	Proliferative: 14 (70%) Secretory: 6 (30%)	Proliferative: 19 (63.3%) Secretory: 11 (36.7%)	0.763

Data are presented as the number of cases or mean ± standard deviation, as appropriate. Comparisons between groups were performed using Student’s *t* test for continuous variables and Fisher’s exact test for categorical variables. No significant differences were observed between the two groups for any of the parameters

Next, we investigated the direct effects of Pro-EGCG on human M-MDSCs using an *in vitro* differentiation model (Additional file 1: Fig. S2, Fig. 5C). Human M-MDSCs were generated from PBMCs and validated for purity prior to functional assays (Additional file 1: Fig. S2). Exposure to Pro-EGCG significantly compromised M-MDSC viability and induced apoptosis in a dose-dependent manner (Fig. 5D, E). Crucially, Pro-EGCG treatment substantially impaired the ability of M-MDSCs to suppress T cell proliferation in co-culture assays, as evidenced by the restoration of T cell CFSE dilution (Fig. 5F). This functional impairment was accompanied by a marked attenuation of the immunosuppressive phenotype, characterized by decreased PD-L1 positivity and diminished production of key suppressive mediators, including ROS, Arg-1, and NO (Fig. 5G–J). These findings were further corroborated by PD-L1 expression profiles in both clinical samples and our murine model. Specifically, circulating M-MDSCs from endometriosis patients exhibited significantly elevated PD-L1 positivity compared with controls (Additional file 1: Fig. S5A), while Pro-EGCG treatment consistently reduced PD-L1^+^ M-MDSC proportions across the BM, PB, and PF in mice (Additional file 1: Fig. S5B). In contrast, the inhibitory effect on PD-L1 expression in murine PMN-MDSCs was not consistently maintained across these compartments (Additional file 1: Fig. S5B).

Together, these results demonstrate that Pro-EGCG acts on M-MDSCs by compromising their survival and dampening their multifaceted immunosuppressive properties, supporting its potential to alleviate the immunosuppressive environment in endometriosis.

### Pro-EGCG disrupts the pathological interaction between M-MDSCs and endometriotic stromal cells

**Fig. 6:**
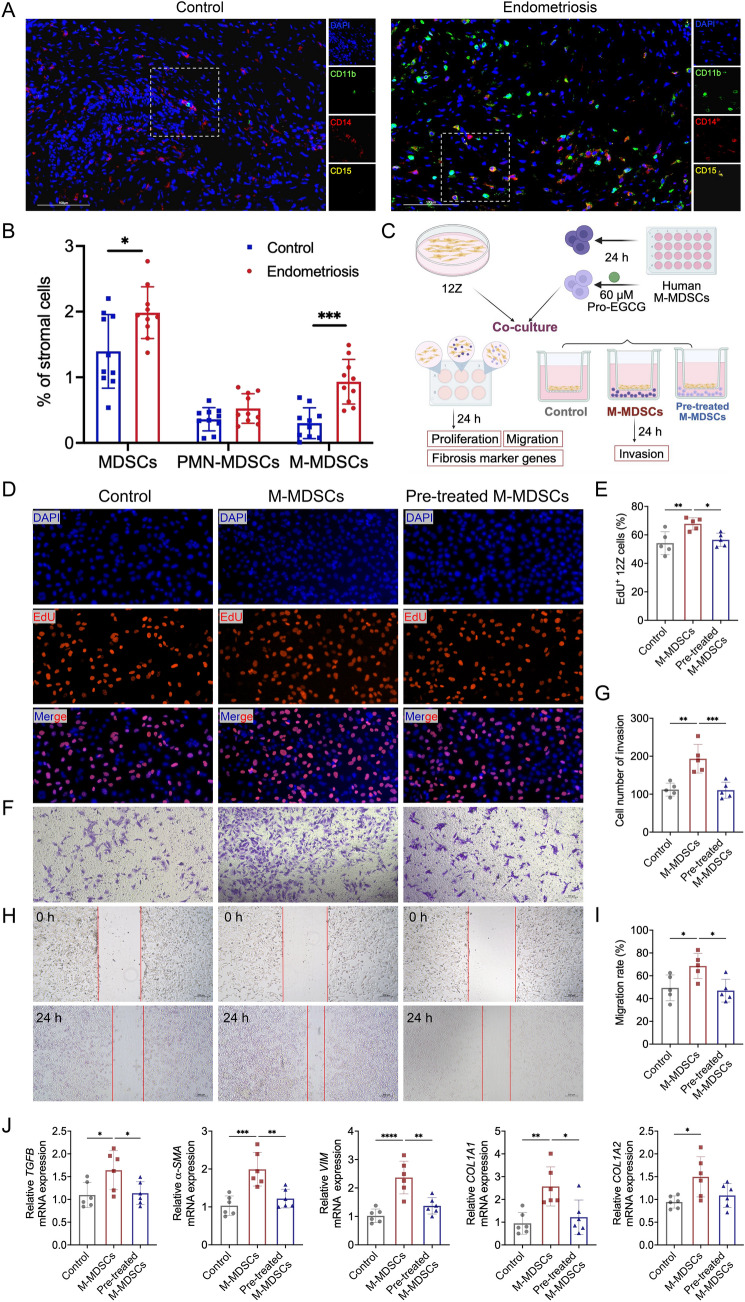
Pro-EGCG disrupts the pathological interaction between M-MDSCs and endometriotic stromal cells. **A**, **B** Representative multiplex immunofluorescence images of ectopic endometrial lesions from patients with endometriosis and eutopic endometrial tissues stained for CD11b (green), CD14 (red), CD15 (yellow), and DAPI (blue). Quantitative analysis shows the proportions of infiltrating CD11b^+^ MDSCs, CD11b^+^CD15^+^ PMN-MDSCs, and CD11b^+^CD14^+^ M-MDSCs relative to stromal cells (n = 10 per group). Scale bar as indicated. **C** Schematic diagram of direct and Transwell co-culture systems designed to evaluate the impact of M-MDSCs on the behavior of endometriotic stromal cells, including proliferation, migration, invasion, and fibrosis-associated gene expression. **D**, **E** Representative images and quantitation of stromal cell proliferation (EdU incorporation). **F**, **G** Representative images and quantitation of stromal cell invasion (Transwell assay). **H**, **I** Representative images and quantitation of stromal migration (scratch wound healing assay) under the indicated co-culture conditions. **J** Quantitative RT-PCR analysis of fibrosis-associated gene expression (*TGFB, ACTA2/α-SMA, VIM, COL1A1, COL1A2*) in stromal cells following co-culture under the indicated conditions. Data are presented as mean ± SD from 5 independent experiments. Statistical significance was determined by unpaired two-tailed Student’s *t* test (**B**) and one-way ANOVA with Tukey’s post-hoc test (**E**, **G**, **I**, **J**). *P < 0.05; ***P < 0.001; ****P < 0.0001

Beyond their immunoregulatory roles, we explored whether M-MDSCs may contribute to lesion progression. Multiplex immunofluorescence revealed a substantial accumulation of CD11b⁺CD14⁺CD15⁻ M-MDSCs in close spatial proximity to stromal cells within human endometriotic lesions (Fig. 6A, B). To explore this interaction, we utilized a co-culture system using human 12Z endometriotic stromal cells in the presence or absence of human M-MDSCs, with or without Pro-EGCG pretreatment (Fig. 6C). Co-culturing with M-MDSCs significantly enhanced the proliferation (Fig. 6D, E), invasiveness (Fig. 6F, G), and migratory capacity (Fig. 6H, I) of 12Z cells. Notably, these pro-endometriotic effects were effectively abolished when M-MDSCs were pre-treated with Pro-EGCG (Fig. 6D–I).

Additionally, co-culturing with M-MDSCs led to a significant upregulation of fibrosis-associated genes in stromal cells at the transcriptional level, including *TGFB, ACTA2 (α-SMA), VIM, COL1A1, and COL1A2* (Fig. 6J). This pro-fibrotic gene expression pattern was markedly attenuated by Pro-EGCG pre-treatment, which significantly suppressed the mRNA levels of *TGFB, ACTA2, VIM,* and *COL1A1* (Fig. 6J).

Collectively, these findings indicate that M-MDSCs promote the proliferative, invasive, and fibrogenic programs of endometriotic stromal cells, and that Pro-EGCG serves as a potent disruptor of these pathogenic cellular interactions.

## Discussion

**Fig. 7:**
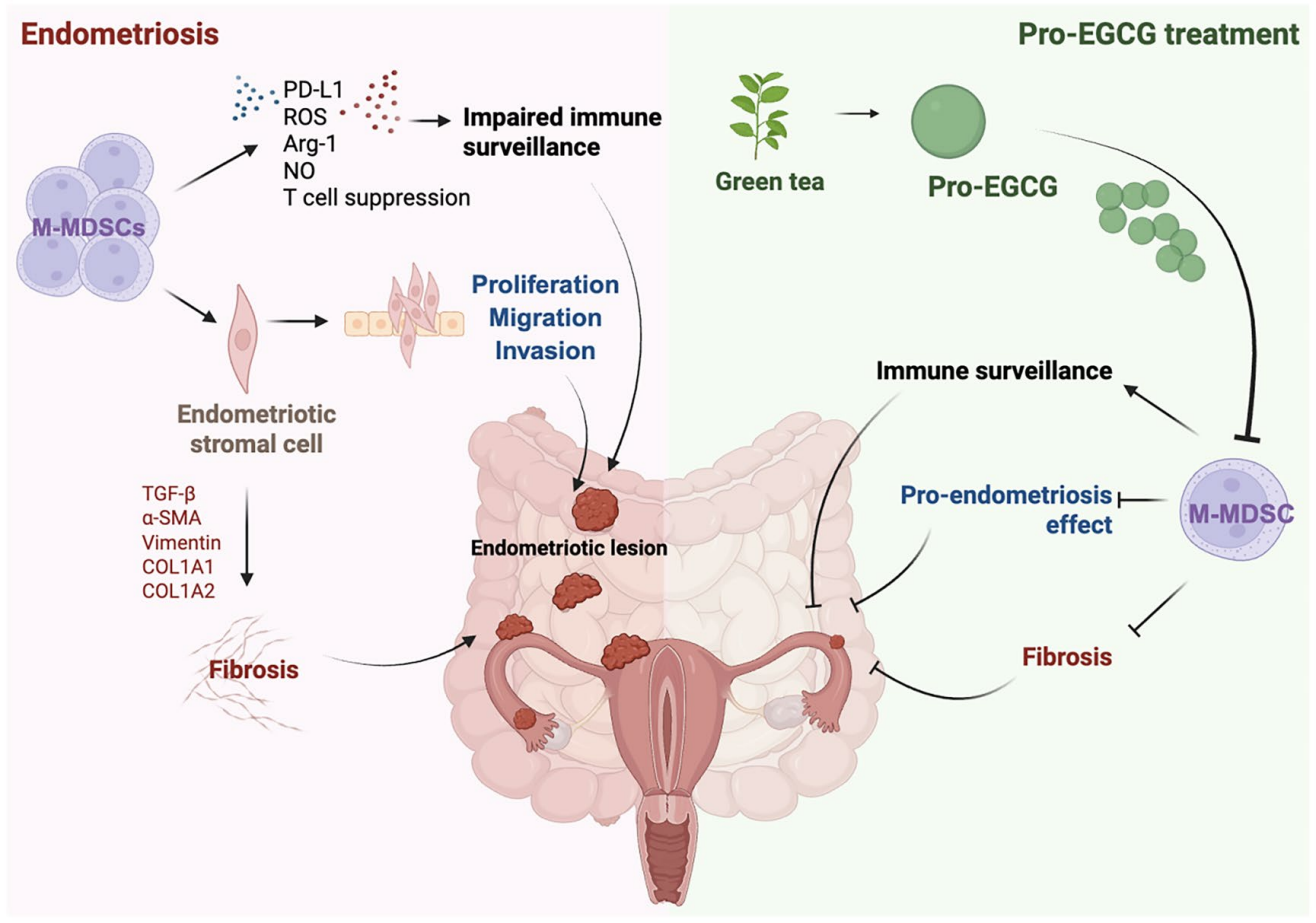
Schematic diagram illustrating the therapeutic mechanism of Pro-EGCG by targeting M-MDSCs in endometriosis. In endometriosis, expansion and infiltration of M-MDSCs facilitate an immunosuppressive environment through increased PD-L1 expression and elevated production of Arg-1, ROS, and NO, which collectively impair immune surveillance. Additionally, M-MDSCs exacerbate the pathological behavior of endometriotic stromal cells by promoting their proliferation, invasiveness, and fibrotic changes. By suppressing M-MDSCs, Pro-EGCG attenuates their immunosuppressive functions and disrupts their pathological interaction with stromal cells, thereby restoring immune surveillance and hindering lesion progression

Emerging evidence has shown that the accumulation of MDSCs contributes to the progression of endometriosis through mechanisms reminiscent of tumor biology, including immune suppression, neovascularization, chronic inflammation, and fibrotic remodeling [14, 27, 28]. In the present study, we demonstrate that Pro-EGCG effectively suppresses the immunosuppressive activity of M-MDSCs and inhibits their capacity to promote endometriotic cell proliferation, invasion, migration, and fibrosis, thereby collectively restraining disease progression (Fig. 7). These findings provide mechanistic evidence supporting the therapeutic effects of Pro-EGCG in endometriosis, shedding light on its broader potential in the treatment of MDSC-driven pathological conditions.

EGCG, the principal polyphenolic component of green tea, has long been recognized for its diverse biological activities, including anti-cancer, anti-angiogenic, and antioxidant effects across a range of disease contexts. However, its clinical translation has been substantially limited by poor bioavailability and rapid degradation, restricting its application largely to nutritional supplementation [29]. To overcome these limitations, a peracetate-protected prodrug, Pro-EGCG, was developed through acetylation of EGCG hydroxyl groups [30], yielding a compound with markedly enhanced stability (approximately six-fold greater than native EGCG) and improved systemic bioavailability [30]. In our previous studies, Pro-EGCG exhibited superior therapeutic efficacy over EGCG in experimental endometriosis, attributable to both improved pharmacokinetic properties and engagement of distinct molecular pathways [8]. Extending these observations, the present study reveals that Pro-EGCG significantly reduces the abundance and functional activity of M-MDSCs in a murine endometriosis model. Importantly, adoptive transfer of M-MDSCs into Pro-EGCG-treated mice partially restored lesion growth and pathological features, providing direct *in vivo* evidence that suppression of M-MDSCs is a critical determinant of Pro-EGCG-mediated therapeutic efficacy.

Accumulating clinical and experimental studies have consistently reported an expansion of MDSCs, particularly M-MDSCs, in the PB, PF, and ectopic lesions of women with endometriosis [11, 31, 32]. Bioinformatic analyses of transcriptomic and single-cell RNA sequencing datasets further support a pronounced enrichment of MDSCs within ectopic lesions compared with eutopic and control endometrial tissues [33]. Our findings align with these observations, demonstrating a predominant expansion of M-MDSCs in circulation and their subsequent infiltration into endometriotic lesions. In line with previous mouse studies, elevated M-MDSC levels in our model were accompanied by sustained lesion survival and high PD-L1 expression, suggesting a disruption of local immune surveillance [11, 31, 34]. Mechanistically, our *in vitro* assays confirmed that human PBMC-derived M-MDSCs possessed potent T-cell suppressive capacity. Furthermore, Pro-EGCG treatment was shown to markedly attenuate their expression of key immunosuppressive markers and mediators, including PD-L1, ROS, Arg-1, and NO. The downregulation of these effector molecules, coupled with the impaired T-cell suppressive capacity observed *in vitro* , suggests that Pro-EGCG re-programs human M-MDSCs toward a less suppressive phenotype [11, 34]. Collectively, these findings suggest that Pro-EGCG may restore impaired immune surveillance in endometriosis by reducing M-MDSC accumulation and dampening their multifaceted immunosuppressive properties.

Beyond their well-characterized role in immune suppression, MDSCs are increasingly recognized as central regulators of the disease microenvironment through non-immune mechanisms that support tissue remodeling and pathological progression [35, 36]. Extensive evidence from oncology has shown that MDSCs promote angiogenesis, enhance malignant cell survival, and drive stromal remodeling and fibrosis within tumor tissues [37–40]. Endometriosis shares multiple pathological hallmarks with cancer, including invasive growth, aberrant angiogenesis, resistance to apoptosis, and progressive fibrotic remodeling. In this context, the positive correlations between lesion-infiltrating M-MDSCs and histopathological indices of proliferation and fibrosis, together with the inverse correlation with apoptosis, indicate that M-MDSC abundance is closely associated with lesion severity. These findings should be interpreted as associative rather than causal. Nevertheless, when considered alongside our previous *in vivo* study showing that anti-Gr-1 antibody-mediated depletion of MDSCs markedly suppressed endometriotic lesion development [11], as well as the present adoptive transfer and *in vitro* co-culture experiments, they support a functionally relevant role for M-MDSCs in facilitating endometriosis progression. Building on our previous work demonstrating the pro-angiogenic role of M-MDSCs in endometriotic lesions [11], the present study provides evidence that M-MDSCs also exacerbate the pathogenic behavior of endometriotic stromal cells. Specifically, M-MDSCs enhanced stromal cell proliferation, migration, and invasion, while robustly inducing a pro-fibrotic gene signature. These findings expand our understanding of M-MDSCs from classical immune suppressors to orchestrators of lesion remodeling. Notably, these pro-endometriotic effects were effectively disrupted by Pro-EGCG treatment, positioning Pro-EGCG as a multifaceted therapeutic agent capable of targeting both immune and stromal components of the lesion microenvironment (Fig. 7). However, it should be acknowledged that the mechanistic evidence presented above is primarily derived from *in vitro* models. The *in vivo *situation is likely more complex, as stromal cells within the endometriotic lesion microenvironment secrete chemokines, including CXCL1, CXCL2, and CXCL5, which recruit myeloid cells through CXCR5, as well as CCL25, which signals through CCR9 [11, 32]. Pro-EGCG may therefore additionally modulate M-MDSC accumulation indirectly by altering the chemokine milieu of the lesion microenvironment. Future studies dissecting the relative contributions of direct and indirect mechanisms *in vivo* would further advance our understanding of Pro-EGCG's immunomodulatory actions in endometriosis.

Targeting MDSCs has emerged as a promising therapeutic strategy in cancer immunotherapy [18, 41]. Multiple approaches have been explored, including pharmacological inhibition of the JAK2/STAT3 signaling axis, induction of MDSC differentiation, and activation of innate immune pathways [14, 42, 43]. However, many of these strategies are associated with systemic toxicity, narrow pharmacologic windows, and limited tolerability, particularly in chronic disease settings [35]. In this context, our study identifies Pro-EGCG as a safe, naturally derived small molecule capable of suppressing M-MDSC expansion and function. Consistent with its improved bioavailability relative to EGCG, Pro-EGCG achieved effective M-MDSC suppression at a substantially lower dose. Whereas EGCG typically requires daily doses exceeding 125–250 mg/kg to reduce MDSC accumulation in breast cancer models [44], Pro-EGCG elicited a robust immunomodulatory effects at a dose of 50 mg/kg without detectable hepatic or renal toxicity. These properties support the potential of Pro-EGCG as a long-term immunomodulatory therapy. Particularly in endometriosis, a chronic condition requiring sustained management, the superior safety profile and lower effective dose of Pro-EGCG offers a more viable clinical path than native EGCG for long-term intervention. Furthermore, given the fundamental role of M-MDSCs in driving pathological progression, these findings may extend the therapeutic utility of Pro-EGCG to other MDSC-driven chronic inflammatory and fibro-proliferative diseases.

Several limitations of the present study should be acknowledged. Although our *in vitro* co-culture systems provide mechanistic insight into interactions between M-MDSCs and endometriotic stromal cells, they cannot fully recapitulate the complexity of the human lesion microenvironment. Additionally, in the adoptive transfer model, M-MDSCs were derived from endometriosis-bearing donors and may therefore have been conditioned by the disease microenvironment. Whether naïve M-MDSCs from healthy hosts exhibit comparable pro-endometriotic activity, and whether the observed effects reflect intrinsic cellular properties or environmentally acquired functional states, remains to be determined in future studies. Moreover, elucidating the precise molecular targets through which Pro-EGCG regulates M-MDSCs remains challenging. M-MDSCs are a rare, heterogeneous, and functionally plastic population, and conventional genetic manipulation approaches, such as lentiviral transfection or nucleic acid electroporation, can perturb their native functional state, altering key properties including chemokine responsiveness and suppressive capacity [13, 45–47]. These inherent biological and technical constraints complicate efforts to define direct binding targets and downstream signaling pathways. Nevertheless, as a small molecule, Pro-EGCG is likely to exert its effects through pleiotropic interactions with multiple molecular targets, rather than a single ‘lock-and-key’ mechanism, consistent with our previous reports [8, 48, 49].

## Conclusion

In conclusion, this study expands the therapeutic framework of Pro-EGCG by identifying MDSCs, particularly M-MDSCs, as a crucial cellular target underlying its efficacy in endometriosis. Our findings provide compelling evidence that targeting MDSC-mediated immune and stromal dysregulation represents a fundamental therapeutic strategy for endometriosis and position Pro-EGCG as a promising multifunctional candidate warranting further clinical investigation.

## Supplementary Information

Additional file 1:  Supplementary figures and tables.

## Data Availability

The datasets supporting the conclusions of this article are included within the article and its additional files.

## Abbreviations

α-SMA: Alpha-smooth muscle actin
ACTA2: Actin alpha 2
ACTB: Beta-actin
ALT: Alanine aminotransferase
Arg-1: Arginase-1
AST: Aspartate aminotransferase
BM: Bone marrow
CCK-8: Cell counting kit-8
CFSE: Carboxyfluorescein succinimidyl ester
DCF-DA: 2′,7′-Dichlorofluorescein diacetate
EGCG: Epigallocatechin-3-gallate
FBS: Fetal bovine serum
GM-CSF: Granulocyte–macrophage colony-stimulating factor
HLA-DR: Human leukocyte antigen–DR isotype
IL-6: Interleukin-6
JAK2/STAT3: Janus kinase 2/signal transducer and activator of transcription 3
M-MDSCs: Monocytic myeloid-derived suppressor cells
MDSCs: Myeloid-derived suppressor cells
NK: Natural killer
NO: Nitric oxide
PB: Peripheral blood
PBMCs: Peripheral blood mononuclear cells
PD-L1: Programmed death-ligand 1
PF: Peritoneal fluid
PMN-MDSCs: Polymorphonuclear myeloid-derived suppressor cells
Pro-EGCG: Peracetylated epigallocatechin-3-gallate
RBC: Red blood cells
ROS: Reactive oxygen species
TGF-β: Transforming growth factor-beta
TUNEL: Terminal deoxynucleotidyl transferase-mediated dUTP nick-end labeling
