# Supplementary material for: Pro-EGCG suppresses endometriosis progression via regulating monocytic myeloid-derived suppressor cells
Source: Chin Med. 2026 Jul 1;21:177. doi: 10.1186/s13020-026-01451-8 (PMC13321424; doi:10.1186/s13020-026-01451-8)
Supplement: Supplementary file 1 — Additional file 1: Supplementary figures and tables. [file 13020_2026_1451_MOESM1_ESM.pdf]

Supplementary Fig. 1

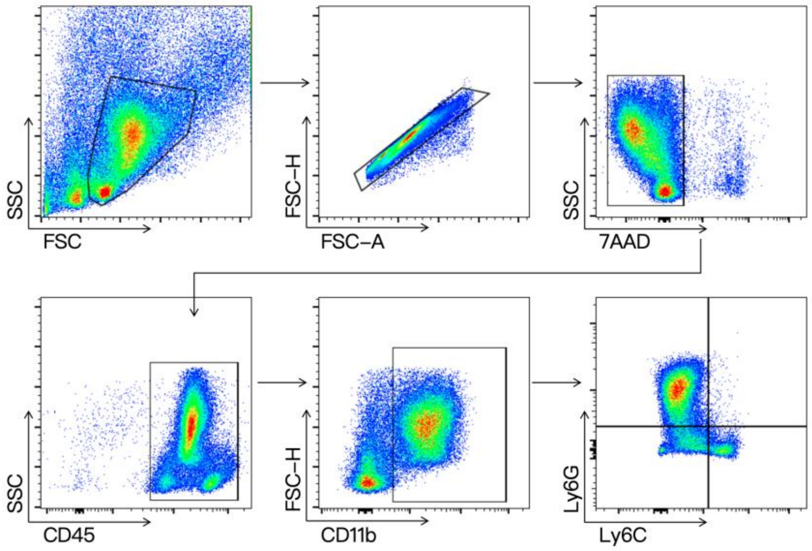

Fig. S1 Gating strategy for identification of MDSC subsets in mouse samples by flow cytometry. Representative flow cytometry plots illustrating the sequential gating strategy used to identify MDSC subsets in murine samples. Cells were first gated based on FSC and SSC to exclude debris, followed by singlet discrimination using FSC-A versus FSC-H. Live cells were selected by exclusion of 7-AAD^{+} events. CD45^{+} leukocytes were subsequently gated, within which CD11b^{+} myeloid cells were identified. PMN-MDSCs and M-MDSCs were defined as CD11b^{+}Ly6G^{+}Ly6C^{-} and CD11b^{+}Ly6G^{-} Ly6C^{+} populations, respectively. This gating strategy was consistently applied to bone marrow, peripheral blood, and peritoneal fluid-derived single-cell suspensions.

Supplementary Fig. 2

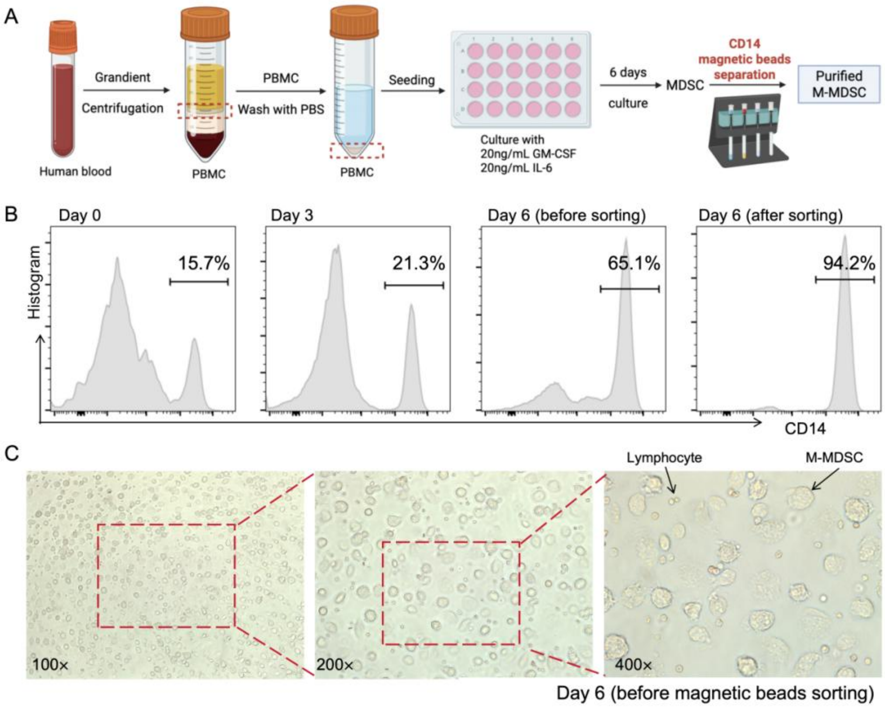

Fig. S2 *In vitro* generation and characterization of human M-MDSCs. A Schematic overview of the protocol used to generate human M-MDSCs from peripheral blood mononuclear cells (PBMCs). PBMCs were cultured with granulocyte-macrophage colony-stimulating factor (GM-CSF) and interleukin-6 (IL-6) for 6 days, followed by $CD14^+$ cell isolation using magnetic beads. **B** Flow cytometric analysis of $CD14^+$ M-MDSCs at day 0, day 3, day 6 before sorting, and at day 6 after $CD14^+$ cell isolation. **C** Representative bright-field microscopy images of cultured cells at day 6 (100×, 200×, and 400× magnification), illustrating the morphology of M-MDSCs and residual lymphocytes.

Supplementary Fig. 3

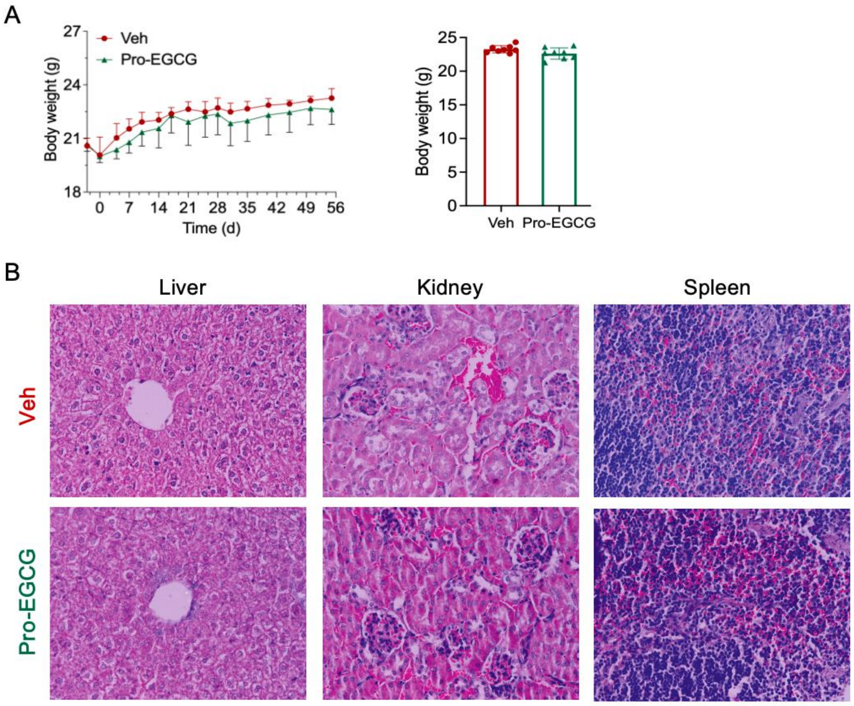

Fig. S3 Pro-EGCG treatment does not induce detectable systemic toxicity in mice. A Body weight monitoring throughout the 8-week experimental period and final body weights at the study endpoint. **B** Representative H&E-stained sections of liver, kidney, and spleen from vehicle- and Pro-EGCG-treated mice. Data are presented as mean ± SD (n = 8 mice per group). Statistical analysis revealed no significant differences between groups.

Supplementary Fig. 4

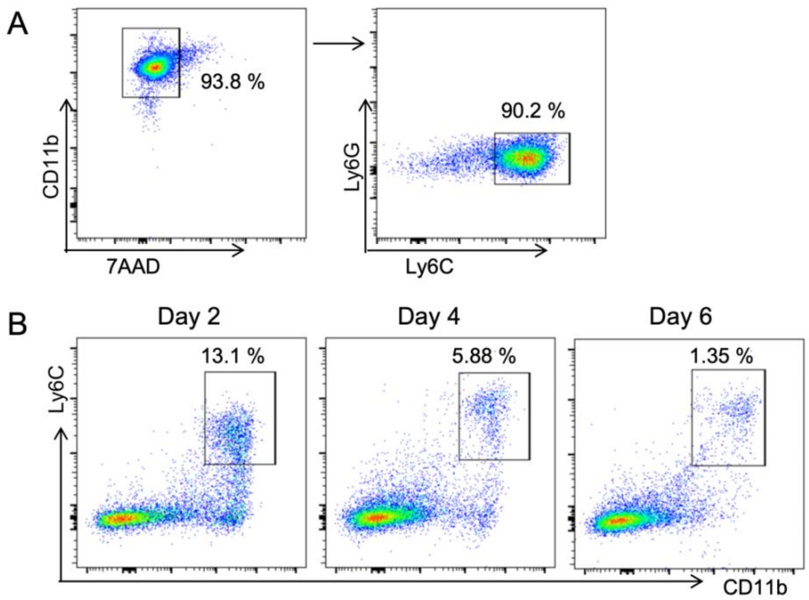

Fig S4 Characterization of M-MDSCs used for adoptive transfer and temporal changes in circulating M-MDSCs following transfer. **A** Purity and viability of sorted M-MDSCs ($CD11b^{+}Ly6C^{+}Ly6G^{-}$) assessed by flow cytometry prior to adoptive transfer. **B** Temporal changes in the frequency of circulating M-MDSCs in peripheral blood of recipient mice at the indicated time points following adoptive transfer.

Supplementary Fig. 5

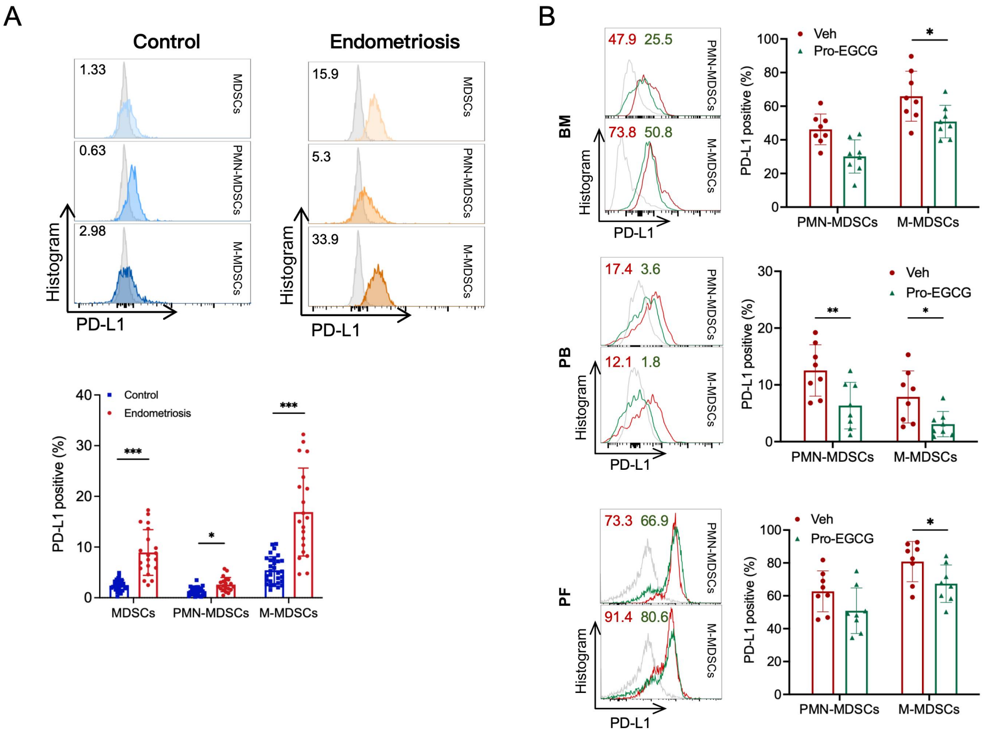

Figure S5. Pro-EGCG suppresses PD-L1 expression on MDSC subsets in clinical samples and the murine model. A Representative histograms and quantification of PD-L1 positivity on circulating MDSCs, PMN-MDSCs, and M-MDSCs from control individuals (n=30) and endometriosis patients (n=20). **B** Representative histograms and quantification of PD-L1 expression on PMN-MDSCs and M-MDSCs in the BM, PB, and PF of vehicle- or Pro-EGCG-treated mice (n = 8 per group). Data are presented as mean ± SD (n=8 mice per group). Statistical significance was determined by unpaired two-tailed Student's t-test. *$P < 0.05$, **$P < 0.01$, ***$P < 0.001$.

Supplementary Table 1. Antibodies used for flow cytometry and multiplex immunofluorescence.

Antibody / Reagent	Company	Catalog Number
**For mouse flow cytometry**		
anti-CD45 APC antibody	BioLegend	103112
anti-CD11b APC/Fire™ 750 antibody	BioLegend	101261
anti-Ly-6G Brilliant Violet 421™ (BV421) antibody	BioLegend	127627
anti-Ly-6C Brilliant Violet 605™ (BV605) antibody	BioLegend	128035
anti-PD-L1 PE/Cy7 antibody	BioLegend	124313
7-AAD Viability Staining Solution	BioLegend	420403
**For human flow cytometry**		
anti-CD45 PE/Cy7 antibody	BioLegend	368532
anti-CD33 BV421 antibody	BioLegend	366621
anti-CD11b Alexa Fluor® 700 (AF700) antibody	BioLegend	301356
anti-HLA-DR APC/Cy7 antibody	BioLegend	307618
anti-CD14 BV510 antibody	BioLegend	301841
anti-CD15 FITC antibody	BioLegend	394706
anti-PD-L1 APC antibody	BioLegend	329708
7-AAD Viability Staining Solution	BioLegend	420403
**For multiplex immunofluorescence staining**		
anti-CD11b antibody	abcam	Ab13357
anti-Ly6C antibody	abcam	AB317272
anti-Ly6G antibody	Biolegend	127601
CD14 (Y0403) Rabbit mAb	Huilanbio	HL18248
CD15 Recombinant Rabbit mAb (S009)	Huilanbio	HL32041

Supplementary Table 2. Sequences of primers used for qRT-PCR

Gene symbol	Gene name	Primer sequence (5' to 3')
**VIM**	Vimentin	Forward: CCTCCGGGAGAAATTGCAGG
		Reverse: GCGTTCAAGGTCAAGACGTG
**ACTA**	Alpha smooth muscle actin	Forward: GTAGCTACCCGCCCAGAAAC
		Reverse: TGACCCATACCGACCATGAC
**TGFB1**	Transforming growth factor beta 1	Forward: GCCTCCTCCTGCCTGTCTG
		Reverse: AGTGCCCAAGGTGCTCAATAAATAG
**COL1A1**	Collagen type I alpha 1 chain	Forward: CTCTGAAGGTCCCCAGGGT
		Reverse: GCAATACCAGGAGCACCATTG
**COL1A2**	Collagen type I alpha 2 chain	Forward: ACTGTAAGAAAGGGCCCAGC
		Reverse: GGGCCAAGTCCAACTCCTTT
**CTGF**	Connective tissue growth factor	Forward: CGCACAAGGGCCTATTCTGT
		Reverse: GAGCACCATCTTTGGCGGT
**FN1**	Fibronectin 1	Forward: CCGCCGAATGTAGGACAAGA
		Reverse: TGTCAGAGTGGCACTGGTAG
**ACTB**	Actin beta	Forward: ACAGAGCCTCGCCTTTGCC
		Reverse: GATATCATCATCCATGGTGAGCTGG
